# Plasma levels of high density lipoprotein cholesterol and outcomes in chronic thromboembolic pulmonary hypertension

**DOI:** 10.1371/journal.pone.0197700

**Published:** 2018-05-29

**Authors:** Ghaleb Khirfan, Vickram Tejwani, Xiaofeng Wang, Manshi Li, Joseph DiDonato, Raed A. Dweik, Nicholas Smedira, Gustavo A. Heresi

**Affiliations:** 1 Department of Internal Medicine, Medicine Institute, Cleveland Clinic, Cleveland, Ohio, United States of America; 2 Division of Pulmonary and Critical Care Medicine, Johns Hopkins Hospital, Baltimore, Maryland, United States of America; 3 Department of Quantitative Health Sciences, Cleveland Clinic, Cleveland, Ohio, United States of America; 4 Department of Cellular and Molecular Medicine, Cleveland Clinic, Cleveland, Ohio, United States of America; 5 Department of Pulmonary and Critical Care Medicine, Respiratory Institute, Cleveland Clinic, Cleveland, Ohio, United States of America; 6 Department of Thoracic and Cardiovascular Surgery, Cleveland Clinic, Cleveland, Ohio, United States of America; Ann and Robert H Lurie Children’s Hospital of Chicago, Northwestern University, UNITED STATES

## Abstract

**Background:**

High Density Lipoprotein Cholesterol (HDL-C) has various anti-inflammatory, anti-atherogenic, anti-oxidant and anti-coagulant properties that improve vascular function. The utility of HDL-C as a biomarker of severity and predictor of survival was described in patients with pulmonary arterial hypertension (PAH). No prior study has assessed the utility of HDL-C in patients with Chronic Thromboembolic Pulmonary Hypertension (CTEPH).

**Objectives:**

We aim to measure HDL-C levels in CTEPH patients and compare it to those in PAH patients and controls and determine HDL-C associations with markers of disease severity, hemodynamics and mortality in CTEPH.

**Methods:**

We retrospectively included patients with CTEPH, identified from the Cleveland Clinic Pulmonary Hypertension Registry. All patients had right heart catheterization (RHC) and imaging studies consistent with CTEPH. We collected demographics, co-morbidities, baseline laboratory data including plasma HDL-C, six-minute walk test (6MWT), echocardiography and RHC. HDL-C levels were compared to a cohort of patients with cardiovascular risk factors and a previously published PAH cohort.

**Results:**

HDL-C levels were available for 90 patients with CTEPH (age: 57.4±13.9 years; female 40%), 69 patients with PAH (age: 46.7±12.8 years; female 90%) and 254 control subjects (age: 56.7±13 years; female 48%). HDL-C levels in CTEPH patients were lower compared to controls and higher compared to PAH patients (median, IQR: CTEPH: 44, 34–57 mg/dl; PAH: 35.3, 29–39 mg/dl; Control: 49, 40–60 mg/dl; p < 0.01 for both pairwise comparisons). In CTEPH, higher HDL-C was associated with decreased prevalence of right ventricular dilation on echocardiography (p = 0.02). 57 patients with CTEPH underwent pulmonary thromboendarterectomy, higher HDL-C was associated with a larger decrement in postoperative pulmonary vascular resistance (PVR) (r = 0.37, p = 0.049). HDL-C was not associated with mortality or other markers of disease severity.

**Conclusions:**

HDL-C levels in CTEPH patients were lower compared to control subjects, but higher compared to PAH patients. Higher HDL-C in CTEPH was associated with less right ventricular dilation and greater decrement in postoperative PVR. These data suggest that HDL-C may be a useful marker of small vessel disease in CTEPH.

## Introduction

Chronic thromboembolic pulmonary hypertension (CTEPH), which is classified as group 4 pulmonary hypertension (PH) in the updated clinical classification of pulmonary hypertension [1], is a relatively rare sequela of pulmonary embolism, with estimated incidence of 0.57% to 3.8% after an episode of acute pulmonary embolism [2, 3]. It is caused by obstruction of the pulmonary vessels and distal small vessel vasculopathy that lead to increase in the pulmonary artery pressure (PAP) and pulmonary vascular resistance (PVR). It has a dismal prognosis without timely surgical intervention, leading to right heart failure and death [4, 5].

While the treatment of pulmonary artery hypertension (PAH) relies mainly on PAH specific therapies, with none of these therapies being curative [6], pulmonary thromboendarterectomy (PTE) in CTEPH provides a curative option with excellent long term outcome in carefully selected patients [5, 7]. For patients who have inoperable disease or who are poor surgical candidates, medical therapy [8] or balloon pulmonary angioplasty [9] have become successful alternate options. This highlights the importance of identifying biomarkers and predictors of outcome in both operable and inoperable CTEPH.

Recent studies have identified high density lipoprotein Cholesterol (HDL-C) as a biomarker of severity and predictor of survival in PAH patients, with lower plasma levels being associated with worse outcome [10–12]. The above observation could be explained by the fact that HDL-C improves endothelial function and protects against atherosclerosis through several mechanisms [13]; it stimulates reverse cholesterol transport [14], has various anti-oxidant, anti-inflammatory [15, 16], and anti-coagulant effects [17, 18]. HDL-C also promotes nitric oxide synthase, which is responsible for the synthesis of the vasodilator nitric oxide [19, 20], and it stimulates the release and increases the half-life of prostacyclin [21–24]. The utility of HDL-C as a biomarker in patients with CTEPH has not been previously studied.

Based on the above observations, we hypothesized that HDL-C is also a mediator in CTEPH, both operable and inoperable disease. In this study we aim to measure plasma HDL-C level in CTEPH patients, compare it to PAH patients and control subjects, ascertain associations with hemodynamics in CTEPH, and determine if HDL-C is associated with mortality.

## Methods

### Study subjects

This retrospective study was approved by the Cleveland Clinic Institutional Review Board (study number: IRB 8097). Written informed consent was waived as the data were analyzed anonymously. Patients were identified from the Cleveland Clinic Pulmonary Hypertension Registry. We retrospectively included patients with CTEPH. All patients had right heart catheterization (RHC) and imaging studies consistent with CTEPH characterized by a mean pulmonary artery (PA) pressure ≥ 25 mmHg, PA occlusion pressure ≤ 15 mmHg and mismatched perfusion defects on lung scan [25]. We used 2 previously published cohorts of patients for comparison; the first cohort included a set of subjects referred to the Preventive Cardiology Section from the Cleveland Clinic, who had been referred for assessment of their cardiovascular risk given the presence of risk factors, the other cohort included patients diagnosed with PAH group 1 [1].

### Laboratory and clinical data

In the subset of patients with CTEPH, we collected data regarding demographics, co-morbidities, body mass index (BMI), smoking status, statin therapy, functional class as determined by the New York Heart Association (NYHA) classification, all-cause mortality, baseline laboratory data including plasma HDL-C, six-minute walk test (6MWT), echocardiography and RHC. For those patients who underwent PTE, results of post-operative RHC were collected and in-hospital mortality was recorded. Correlation between plasma HDL-C levels and markers of disease severity were assessed, including total distance walked during six minute walk test (6MWT), heart rate recovery after 6MWT, echocardiographic markers of disease severity [right ventricular systolic pressure (RVSP), right ventricular dilation, right atrial dilation and the presence of pericardial effusion], N-terminal pro b-type natriuretic peptide (NT-proBNP) and hemodynamic measures including mean pulmonary artery pressure(mPAP), cardiac output, cardiac index, pulmonary vascular resistance(PVR) and total pulmonary resistance(TPR).

### Statistical analysis

The study variables were described using mean, standard deviation, median, 25% quartile, 75% quartile and range for continuous variables and counts and percentage for categorical variables. The study group was divided into two groups based upon the outcome. Categorical variables were compared using the Pearson’s chi-square test whereas continuous variables were compared using the two-sample independent t-test. For survival analysis, proportionality tests were performed to check the assumption of survival analysis. Univariate Cox proportional hazards models were performed. Correlations among continuous variables were checked and the variables that might cause collinearity problems were dropped. Stepwise method was used for variable selections in multivariate survival analysis.

For comparison among the three groups of patients (CTEPH, PAH and control group) ANOVA was performed to compare the continuous variables, Chi-square tests were used to compare the categorical variables and Fisher’s exact tests were used when one or more of the cells had an expected frequency of five or less. Pairwise comparisons including post hoc tests were performed. Tukey-Kramer adjustment was used for the continuous variables and Fisher’s exact test with permutation was used for the categorical variables. All analyses were performed by using SAS 9.4 software (SAS Institute, Cary, NC). The level of statistical significance was set at p < 0.05 (two tailed).

## Results

A total of 223 patients with CTEPH were identified from the Cleveland Clinic Pulmonary Hypertension Registry; these were seen for the first time in our clinic between March 1998 and January 2016. Patients with no HDL-C measurements were excluded; we included a total of 90 patients with CTEPH (mean age ±SD: 57.4±13.9 years; female 40%), 69 patients with PAH (mean age: 46.7±12.8 years; female 90%) and 229 control subjects (mean age ±SD: 56.7±13 years; female 47.6%). Table 1 shows baseline characteristics for the group of patients with CTEPH. In pairwise comparison, there were no significant differences in age, gender, prevalence of diabetes mellitus (DM), hypertension (HTN) or smoking status between patient with CTEPH and the control group of subjects with cardiovascular risk factors. As expected, patients with PAH were significantly younger (p<0.0001), had more females (p<0.0001) and decreased prevalence of DM (p<0.0001), HTN (p<0.025) and coronary artery disease (CAD) (P< 0.001) compared to the other 2 groups. Table 2 shows baseline characteristics for the three groups of patients.

**Table 1:** Baseline characteristics for the group of patients with CTEPH.

Variable	CTEPH (n = 90)
Age, yr	57.4 (13.9)
Female gender, n (%)	36 (40)
BMI(kg/m^2^)	32.5 (15.6)
DM, n (%)	18 (20)
HTN, n (%)	44 (48)
Dyslipidemia, n (%)	27 (30.3)
Hypothyroidism, n (%)	7 (7.8)
CAD, n (%)	21 (23)
Smoker, n (%)	11 (12.2)
Statin Therapy, n (%)	18 (20.2)
DM medications, n (%)	11 (12.2)
Beta Blockers, n (%)	32 (35.6)
Thyroid Replacement Therapy, n (%)	6 (6.7)
Corticosteroids, n (%)	6 (6.7)
6MWT	
Distance walked (m)	324.9 ± 171.4
HDL-C (mg/dL)	44.7 ± 15.5
LDL-C (mg/dL)	89.5 ± 39
NT-pro BNP (pg/ml)	3205 ±7027
RHC	
RA pressure (mmHg)	10.2 ± 6.9
Mean PAP (mmHg)	46.0 ± 13.6
CO(L/min) by thermodilution	5.2 ± 1.8
CI (L/min/m2) by thermodilution	2.5 ± 0.8
PVR (Wood units)	7.5 ± 3.8

**Definition of Abbreviations**: BMI: body mass index, CAD: coronary artery disease, CI: cardiac index, CO: cardiac output, DM: diabetes mellitus, HTN: hypertension, NT-pro BNP: N-terminal **pro** B-type natriuretic peptide, PAP: pulmonary artery pressure, PVR: pulmonary vascular resistance, RA: right atrial, RHC: right heart catheterization, 6MWT: six-minute walk test. Data expressed as mean ± SD unless otherwise indicated.

**Table 2:** Patients’ baseline characteristics and HDL-C levels in the 3 group of patients: CTEPH patients, pulmonary arterial hypertension (PAH) patients and control subjects.

Variable	CTEPH (N = 90)	PAH (N = 69)	Control (N = 229)	p value
**Age**				<0.0001^a^
Mean (SD)	57.4 (13.9)	46.7 (12.8)	56.7 (12.9)	
Median	58.0	47.0	58	
**Gender**				<0.0001^b^
F	36 (40.0%)	62 (89.9%)	109 (47.6%)	
M	54 (60.0%)	7 (10.1%)	120 (52.4%)	
**HDL-C (mg/dL)**				<0.0001^a^
Mean (SD)	44.7 (15.5)	35.2 (11.5)	52.9 (19.7)	
Median	44.0	35.3	49.0	
Q1, Q3	34.0, 57.0	29.2, 39.0	40.0, 60.0	
**Diabetes Mellitus**				<0.0001^c^
No	72 (80.0%)	69 (100.0%)	184 (80.3%)	
Yes	18 (20.0%)	0 (0.0%)	45 (19.7%)	
**Hypertension**				<0.0001^b^
No	46 (51.1%)	54 (78.3%)	106 (46.3%)	
Yes	44 (48.9%)	15 (21.7%)	123 (53.7%)	
**Coronary artery disease**				<0.0001^c^
No	69 (76.7%)	66 (95.7%)	144 (62.9%)	
Yes	21 (23.3%)	3 (4.3%)	85 (37.1%)	
**Smoker**				0.4870^b^
No	79 (87.8%)	59 (85.5%)	207 (90.4%)	
Yes	11 (12.2%)	10 (14.5%)	22 (9.6%)	

p-values:

^a^ = ANOVA,

^b^ = Pearson’s chi-square test,

^c^ = Fisher’s Exact test.

### Baseline HDL-C

**Fig 1:**
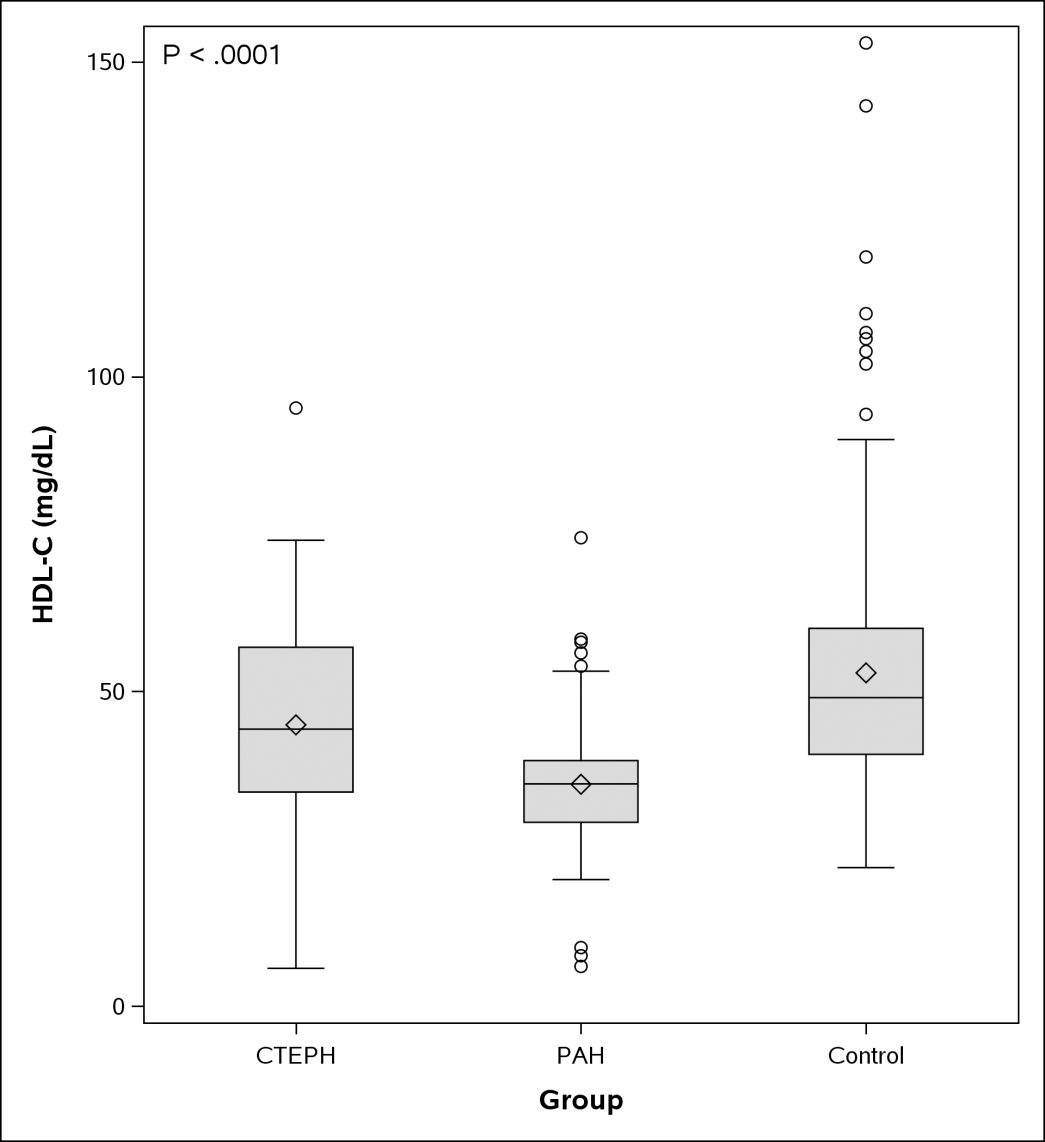
Mean plasma HDL-C (mg/dL) in 3 groups of patients (CTEPH, PAH and control group). HDL-C in CTEPH patients is lower compared to control subjects and higher compared to PAH patients. (Mean± SD): CTEPH: (44.7 ± 15.5 mg/dL); PAH: (35.2±11.5 mg/dL); Control: (52.9 ±19.7 mg/dL); p < 0.0001 for the ANOVA test that was performed to compare mean HDL-C among the three groups of patients. P values for post hoc pairwise comparisons were: CTEPH vs PAH: <0.001, CTEPH vs control: <0.001, PAH vs control: <0.001. Circle symbol represents outliers, diamond symbol represents mean HDL-C values, error bars showing SD of the mean.

HDL-C levels in CTEPH patients were significantly lower compared to control subjects and higher compared to PAH patients (median, interquartile range: CTEPH: 44, 34–57 mg/dl; PAH: 35.3, 29–39 mg/dl; Control: 49, 40–60 mg/dl; p < 0.01, for both pairwise comparisons). Fig 1 shows differences in plasma HDL-C level in the three groups of patients.

A larger percentage of patients in the control group were on statin therapy compared to the other 2 groups (Control: 46.3%, CTEPH: 20.2%, PAH: 11.6%, p < 0.001). HDL-C levels within each group of patients (CTEPH, PAH and control) were not affected by being on statin therapy (Mean HDL-C levels in those on statin therapy versus those not on statin were (CTEPH: 44.5 vs 45.1 mg/dL, p = 0.89, PAH: 34.1 vs 35.4 mg/dL, p = 0.77, control: 50.8 vs 54.8 mg/dL, p = 0.13), respectively. In addition, comparing HDL-C levels among the three groups of patients when adjusting for statin therapy using multiple linear regression model showed that HDL-C levels were still significantly different among these three groups (p<0.0001).

### Correlation between plasma HDL-C and markers of disease severity

**Fig 2:**
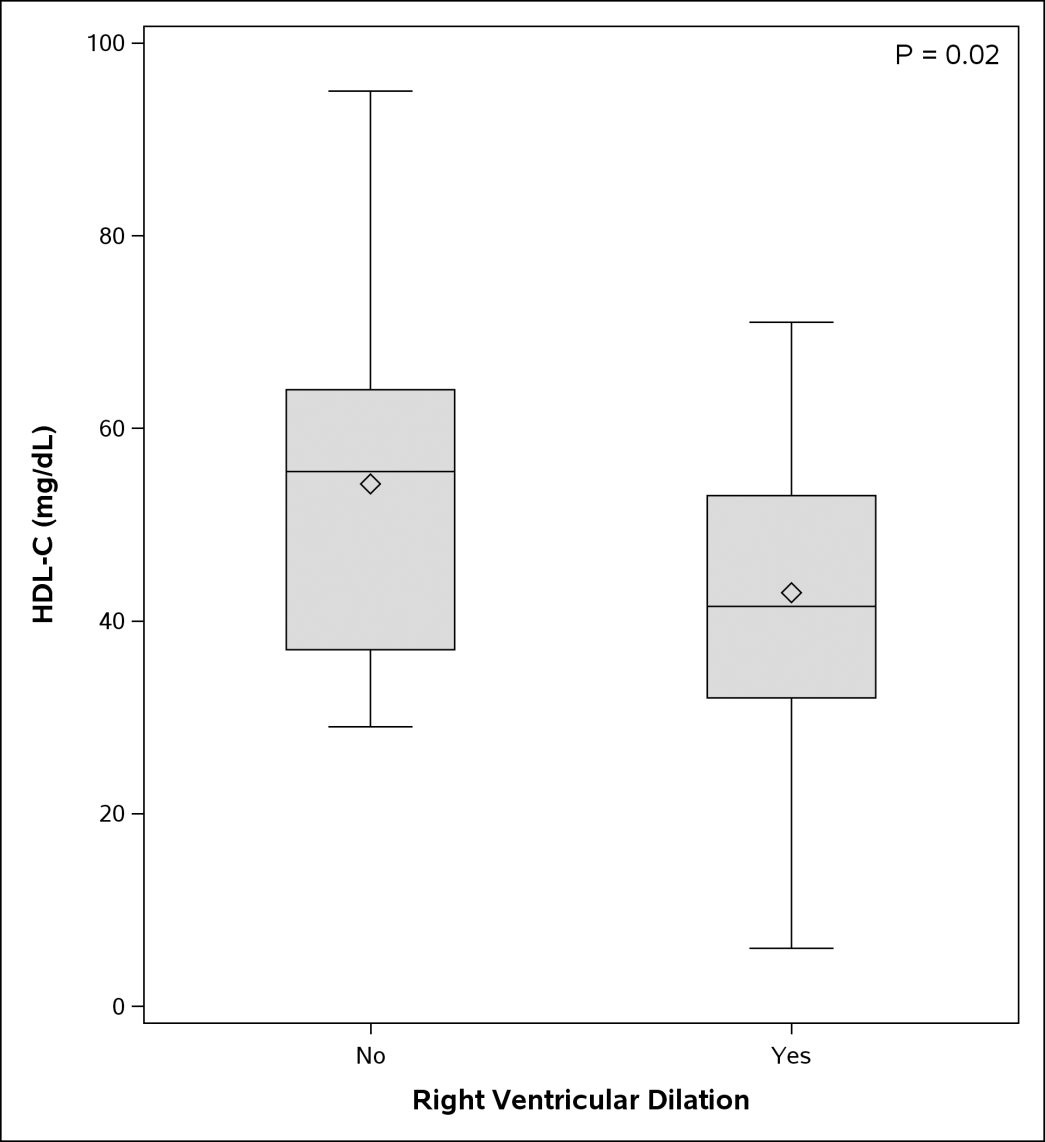
Association between plasma HDL-C (mg/dL) and presence of right ventricular dilation on echocardiography. Univariate logistic regression was performed to assess association. Higher plasma HDL-C was associated with decreased prevalence of right ventricular dilation on echocardiography (p = 0.02). Diamond symbol represents mean HDL-C values.

Higher plasma HDL-C was associated with decreased prevalence of right ventricular dilation on echocardiography (p = 0.02) (Fig 2), with right ventricular size assessment being based on both qualitative (visual assessment) and quantitative (diameter > 41 mm at the base in right ventricular focused view) measures [26]. There were no other statistically significant associations between HDL-C level and other markers of disease severity. Table 3 shows association between plasma HDL-C and markers of disease severity in patients with CTEPH.

**Table 3:** Correlation between plasma HDL-C levels and markers of disease severity in patients with CTEPH. Pearson Correlation Coefficients and P values are shown.

Markers of disease severity	variable	N	Pearson Correlation Coefficients	P value
Total distance walked during 6MWT	HDL-C	64	0.11229	0.3770
Heart rate recovery	HDL-C	42	-0.13391	0.3978
RVSP	HDL-C	86	-0.08195	0.4532
NT-proBNP	HDL-C	47	-0.00183	0.9903
**Hemodynamics**				
Mean right atrial pressure	HDL-C	72	-0.17008	0.1532
mPAP	HDL-C	81	-0.19751	0.0772
CO (thermodilution)	HDL-C	68	0.10583	0.3904
CI (thermodilution)	HDL-C	68	0.20656	0.0910
PVR	HDL-C	63	0.04478	0.7275
TPR	HDL-C	67	-0.07594	0.5414

**Definition of Abbreviations**: CO: cardiac output, CI: cardiac index, mPAP: mean pulmonary artery pressure, NT-proBNP: N-terminal pro b-type natriuretic peptide, PVR: pulmonary vascular resistance, RVSP: right ventricular systolic pressure, TPR: total pulmonary resistance.

**Fig 3:**
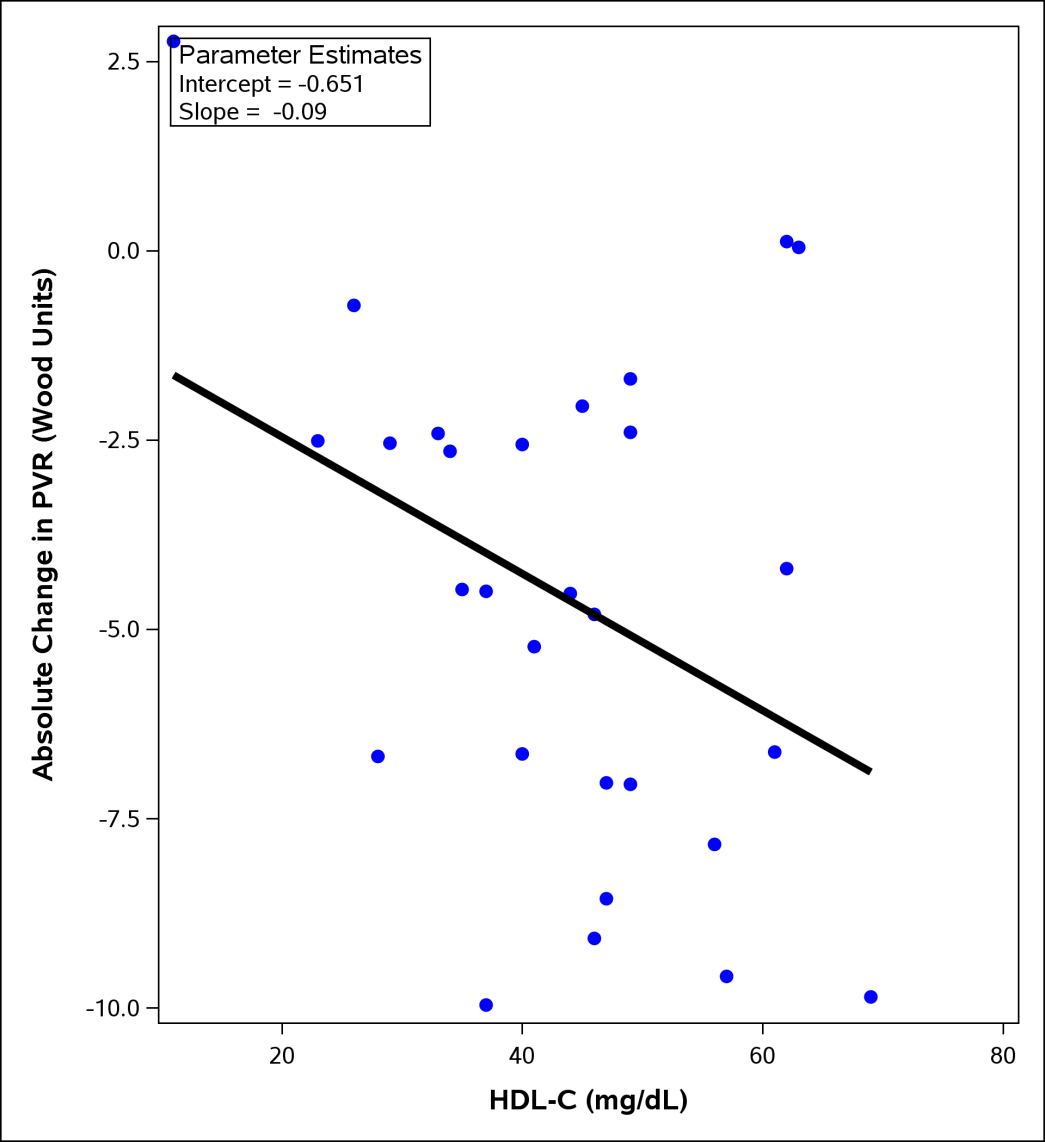
Association between plasma HDL-C (mg/dL) and absolute change in pulmonary vascular resistance (PVR) post pulmonary thromboendarterectomy (PTE). Higher baseline HDL-C was associated with a larger decrease in PVR post PTE (r = 0.37, p = 0.049).

A total of 57 patients with CTEPH underwent PTE. Higher baseline HDL-C in these patients was associated with a larger decrease in postoperative PVR (r = 0.37, p = 0.049) (Fig 3).

### Association between baseline HDL-C and survival

The median follow up length for survival analysis was 1039 days. In the 90 patients with CTEPH, a total of 24 patients died at the time of survival analysis (July 2016). 57 patients underwent PTE, 14 of those died and 2 cases of these deaths were in hospital mortality, defined as death during the same hospitalization for the PTE surgery. Survival analysis using “last known alive date” and logistic regression adjusted for age, gender and BMI for all-cause mortality for the 90 patients with CTEPH, and in hospital mortality showed no association between baseline HDL-C level and mortality.

## Discussion

We found that plasma HDL-C is low in CTEPH, and that lower HDL-C is associated with increased prevalence of right ventricular dilation and blunted PVR improvement after pulmonary endarterectomy.

Numerous metabolic and molecular derangements have been described in PAH patients, including abnormalities in insulin resistance [27], systemic inflammation [28] and energy expenditure [27], with the metabolic theory in PAH previously proposed [29]. Recent studies had shown that HDL-C is lower in patients with PAH, with HDL-C being an independent prognostic factor and marker of disease severity [10–12]. The role HDL-C plays in PAH and its association with survival could be explained by its anti-inflammatory [15, 16], anti-coagulant [17, 18],and endothelial dysfunction attenuating properties [13, 30]. Increasing available prostacyclin [21–23] and promoting nitric oxide synthase by HDL-C [19, 20] are other plausible mechanisms for the role HDL-C plays in PAH, as unbalances in these pathways are implicated in the pathophysiology of PAH, with PAH patients being found to have lower levels of nitric oxide and prostacyclin [31, 32]. CTEPH is caused by obstruction of pulmonary vessels with thromboembolic material due to non-resolution of the thrombi and secondary distal small vessel vasculopathy [33]. Numerous mechanisms had been proposed to explain the non-resolution of thrombotic material in certain patients with pulmonary embolism including abnormal fibrinogen, systemic inflammation and immunological alterations [34]. The small vessel vasculopathy in CTEPH is characterized by lesions typical for the plexiform lesions seen in PAH [35] and it plays a major role in the elevated pulmonary vascular resistance (PVR) observed in CTEPH [34]. The similarity between the small vessel vasculopathy in CTEPH and vascular lesions in PAH, and the prior studies showing abnormalities in immunological response and systemic inflammation in both CTEPH and PAH led us to the hypothesis that abnormalities exist in HDL-C levels in CTEPH patients and that HDL-C might be a marker of small vessel disease in these patients. Indeed, HDL-C levels were lower in CTEPH when compared to controls with similar age, gender and co-morbidities. Low HDL-C correlated with right ventricular dilation. Furthermore, low baseline plasma HDL-C levels were associated with lesser reductions in postoperative PVR in patients who underwent pulmonary thromboendarterectomy. Predicting post-operative outcome in CTEPH is of paramount importance in selecting patients for surgery. Elevated PVR after surgery is the most important predictor of both operative and long-term mortality [5, 36]. In fact, patients with severely elevated PVR that is out of proportion to the thrombotic burden are often inoperable. This decision making remains largely subjective and is driven by expert opinion. Thus, objective parameters would be highly useful. Simple pre-operative non-invasive biomarkers of small vessel vasculopathy in CTEPH, such as plasma HDL-C, offer an opportunity in improve patient selection. Metabolic dysregulation in CTEPH has been previously suggested by Richter et al [37]. These investigators studied the utility of glycosylated hemoglobin A1C (HbA1C) in operable CTEPH patients, baseline HbA1C levels were associated with baseline cardiac index, right atrial pressure, peak oxygen uptake and change in total distance walked during 6MWT post PTE [37]. Elevated HbA1c suggests the presence of insulin resistance and the metabolic syndrome, conditions where low HDL-C is a prominent feature. Our study suggests that HDL-C might be of utility in predicting hemodynamic outcome post PTE in operable CTEPH patients. Other blood biomarkers have been previously found to be of potential value. For example, heart-high type fatty acid-binding protein (H-FABP) concentrations are associated with lower probability of event free survival post PTE [38] and NT-proBNP was found to be associated with survival and able to predict hemodynamic outcome post PTE [39].

PAH patients had lower HDL-C than CTEPH, even though they are younger, had more females and decreased prevalence of co-morbidities. We did not find an association between HDL-C and most markers of CTEPH disease severity or long-term survival. The association with postoperative PVR was weak. While this could be related to low power, this also suggested that that the role HDL-C plays in PAH is more prominent and not totally generalizable to other groups of PH. This is not surprising, since PAH is defined by the presence of small vessel disease. Nonetheless, this strengthens the argument that low HDL-C level could be a marker of microscopic vasculopathy in CTEPH. Larger studies are needed to make more conclusive statements.

### Limitations

Our study has several limitations; the retrospective design, single institution setting and the small sample size could be responsible for the inability to detect association between HDL-C and mortality and other markers of disease severity. A larger percentage of subjects in the control group of patients with cardiovascular risk factors were on statin therapy, although this could be partly responsible for the higher HDL-C in these patients compared to the CTEPH group, our ancillary analysis showed that HDL-C levels within each group of patients (CTEPH, PAH and control) were not affected by being on statin therapy and that HDL-C levels were significantly different among the three groups even after adjusting for statin therapy.

Many studies have linked insulin resistance and the metabolic syndrome to PAH [27, 40–43], the lack of data in our study on insulin levels, fasting blood glucose and hemoglobin A1C level and other lipoproteins could suggest that the observed finding of association between HDL-C in CTEPH with larger decrease in PVR post PTE and decreased prevalence of right ventricular dilation on echocardiography could be related to insulin resistance.

### Conclusions

Plasma HDL-C in CTEPH patients is lower compared to control subjects, but higher compared to PAH patients. Higher HDL-C in CTEPH patients was associated with less right ventricular dilation and greater decrease in postoperative PVR. These data suggest that plasma HDL-C may be a useful marker of small vessel disease in CTEPH. Further studies are needed to assess the utility of HDL-C in CTEPH patients.
